# Efficacy and safety of the new biopsy strategy combining 6-core systematic and 3-core MRI-targeted biopsy in the detection of prostate cancer: Study protocol for a randomized controlled trial

**DOI:** 10.3389/fsurg.2022.1058288

**Published:** 2023-01-06

**Authors:** Chichen Zhang, Xiang Tu, Jindong Dai, Xingyu Xiong, Diming Cai, Ling Yang, Mengni Zhang, Shi Qiu, Tianhai Lin, Zhenhua Liu, Lu Yang, Qiang Wei

**Affiliations:** ^1^ Department of Urology, Institute of Urology, West China Hospital, Sichuan University, Chengdu, China; ^2^ Department of Ultrasound, West China Hospital, Sichuan University, Chengdu, China; ^3^ Department of Radiology, West China Hospital, Sichuan University, Chengdu, China; ^4^ Department of Pathology and Laboratory of Pathology, State Key Laboratory of Biotherapy, West China Hospital, Sichuan University, Chengdu, China

**Keywords:** transrectal ultrasound, systematic biopsy, targeted biopsy, multiparametric magnetic resonance imaging, prostate cancer

## Abstract

**Background:**

Recent EAU guideline strongly recommended combined targeted biopsy (TBx) with systematic biopsy (SBx) for biopsy naïve patients with suspected multiparametric magnetic resonance imaging (mpMRI) lesions; However, the clinical goal is to find out how to determine the optimal SBx and TBx cores for biopsy in order to maximize the detection of csPCa and minimize the associated defects. This study aims to assess the efficacy and safety of the new biopsy strategy combining 6-core systematic and 3-core MRI- TBx compared to 12-core systematic and 3-core MRI-TBx strategy.

**Methods:**

This is a single-center, prospectively randomized controlled clinical trial. 280 men meeting inclusion criteria will be recruited and will be randomly allocated to either 6-core systematic plus 3-core MRI-TBx group (Group A) or 12-core systematic plus 3-core MRI-TBx group (Group B). The primary outcome compares the detection rate of PCa and clinically significant prostate cancer(csPCa) between group A and group B. The secondary outcomes compare the participant-reported pain score immediate post biopsy using pain measurement scale; proportion of men with post-biopsy complications and adverse events (Time frame: 7 days post biopsy, 30 days post biopsy); proportion of the men who undergo radical prostatectomy and have cancer upgraded histopathology from the biopsy to the radical prostatectomy.

**Results and Discussion:**

A new biopsy strategy should be developed with the goal of minimizing procedure invasion, our study will provide the results of efficacy and safety of the new biopsy strategy (6-core systematic and 3-core MRI-TBx) in biopsy naïve men with suspicious mpMRI lesion in comparison with 12-core systematic and 3-core MRI-TBx.

**Trial registration:**

Chinese Clinical Trial Registry, ChiCTR2200056437; http://www.chictr.org.cn/edit.aspx?pid=151413&htm=4

## 1. Introduction

Prostate cancer (PCa) is one of the most diagnosed tumor types among male worldwide, with an estimate of 268,490 new cases and 34,500 deaths in the United States in 2022 (1). Nowadays, for the detection of PCa in patients with a raised prostate-specific antigen (PSA) level, a standard 10–12 cores transrectal ultrasound-guided (TRUS) systematic biopsy (SBx) of the prostate is generally recognized (2). However, this approach might underdetect the clinically significant prostate cancers (csPCa) and overdetect the clinically insignificant cancers.

When the multiparametric magnetic resonance imaging (mpMRI) is widely applied, with the prostate imaging-reporting and data system (PI-RADS) version 2.0 developed in 2015 (3), MRI-targeted biopsy (TBx) has now been accepted and recognized because of its important value in the diagnosis of PCa, especially csPCa (4). In 2018, the PRECISION trial demonstrated that performing four TBx cores for lesions with positive mpMRI (PI-RADS >2) would possibly reduce overdiagnosis and subsequent overtreatment of clinically insignificant cancers while improving the detection of csPCa when compared to a standard 10– 12-core TRUS SBx (5). Though TBx has some benefits, this approach alone may also cause missed diagnosis of PCa, thus result in delayed treatment (6, 7). The 2021 European Association of Urology (EAU) guideline strongly recommended combining TBx with SBx for biopsy naïve patients with suspected mpMRI lesions (2).

However, the optimal cores for the combined systematic and TBx have not been fully investigated. In the era of mpMRI, our previously study found that for suspected mpMRI lesions, the third targeted core can increase the detection rate of csPCa by 11.5% (13.5–26.7%) based on the first two cores; however, the incremental value of adding the fourth or the fifth core was only 6.0% (range: 4.7%–6.9%) and 4.1% respectively (8). Song et al. also supported that a 3-core TBx per lesion was suitable during transperineal MRI ultrasound fusion biopsy. It is worthy of note that currently the SBx still applied the traditional scheme of 10- to 12 cores while adding to the combined biopsy strategy, which might overlap the mpMRI lesions and lead to the unnecessary repeat biopsies. Using as few biopsy cores as possible is likely to reduce procedure time and pathologists' workload and minimize patient discomfort as well as the incidence of complications (9). Therefore, the clinical goal is to figure out how to determine the optimal systematic and TBx cores for the current combined approach, which can maximize the detection rate of csPCa while minimizing the associated defects, especially in the setting of transperineal biopsy under local anesthesia.

Thus, this prospectively randomized controlled trial (RCT) aims to explore the efficacy and safety of the new biopsy strategy combining 6-core systematic and 3-core MRI-TBx compared to 12-core systematic and 3-core MRI-TBx. We will indicate whether 6-core systematic plus 3-core MRI-targeted prostate biopsy could be performed, thus, decreased SBx cores in the detection of csPCa.

## 2. Methods and materials

### 2.1. Ethics and dissemination

The study protocol has obtained the approval of biomedical ethics committee of West China Hospital (No. 2021-1649). The clinical trial registration has been completed at Chinese Clinical Trial Registry (No. ChiCTR2200056437). Participants will be required to provide the written informed consent forms to investigators before they are enrolled in the study. And they will also receive follow-ups from the investigators. The results of this study will be reported through national and international papers or conferences.

### 2.2. Study design, randomization, and blinding

**Figure 1:**
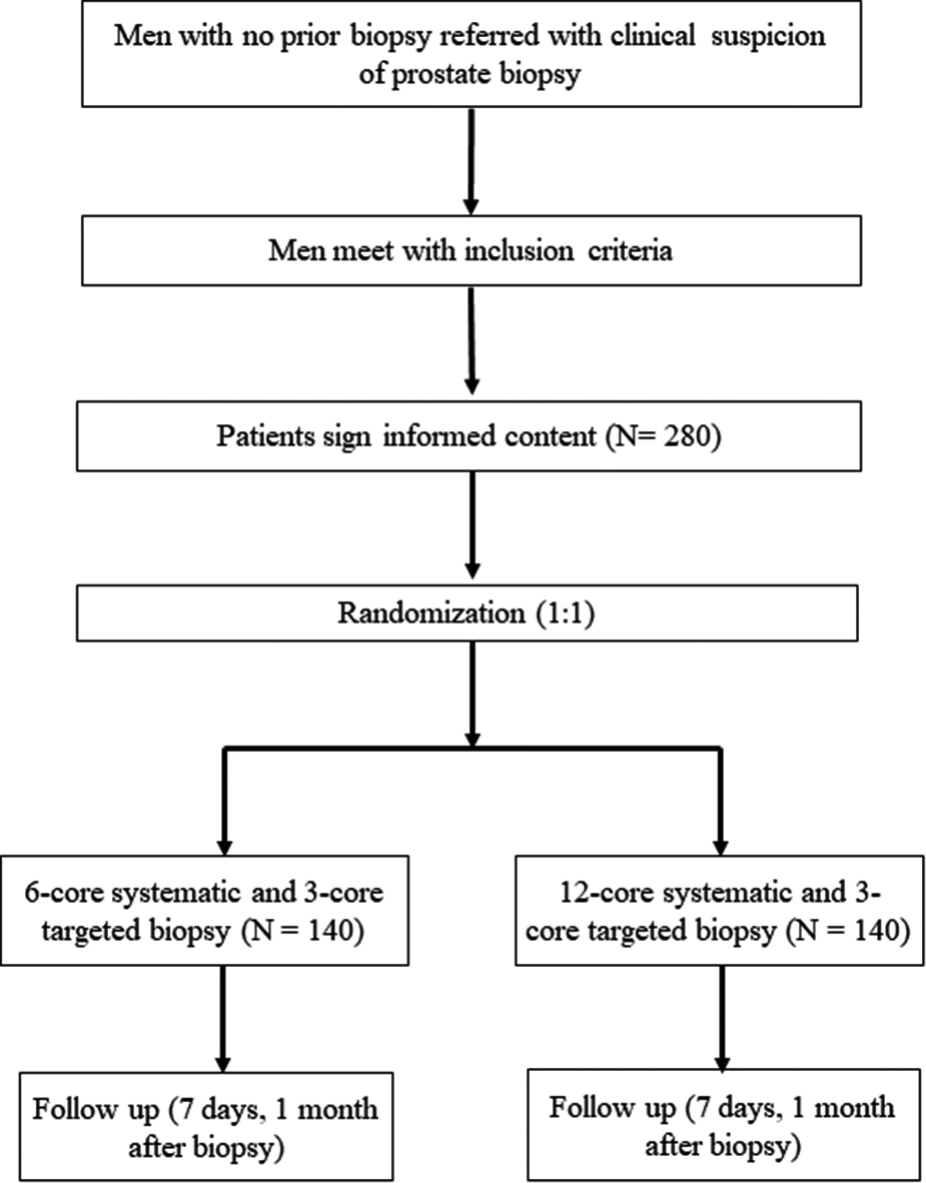
Patients selection flowchart.

Our protocol is a single-center, prospective RCT. Patients' demographic, clinical, and laboratory data will be obtained prospectively from electronic medical records of West China Hospital. We used a computer-generated random number to establish the randomization sequence. Eligible participants will be randomly allocated to either 6-core systematic plus 3-core MRI-TBx group (Group A) or 12-core systematic plus 3-core MRI-TBx group (Group B) with a ratio of 1:1 (Figure 1). In our trial, the urologist will not be blinded to any results because they will use the clinical and MRI findings to make clinical decisions and perform prostate biopsies.

### 2.3. Study participants

Patients will be considered eligibly recruited if they fulfill the following inclusion criteria: (1) Men ≥18 years old with clinical suspicion of PCa (i.e., abnormal digital rectal examination or elevated PSA, PSA ≥ 4 ng/ml); (2) serum PSA level ≤20 ng/ml, clinical stage ≤cT2; (3) Prebiopsy mpMRI performed within 3 months and patients with only solitary MRI lesion (maximal diameter <2 cm) on mpMRI (PIRADS score >2). The exclusion criteria are as follow: (1) men with previous prostate biopsy; (2) prior treatment for prostate; (3) mpMRI done elsewhere; (4) any contraindications for biopsy, such as coagulation disorders, severe internal and external hemorrhoids; (5) patients not willing to write informed consent. Participants can stop participating the study at any time for any reason. Data until the time of withdrawal can be included in the study.

### 2.4. Interventions and outcomes

#### 2.4.1. Multiparametric MRI procedures

Multiparametric MRI will be carried out using a 3.0 T MRI system (Skyra, Siemens, Germany or GE Healthcare, USA). Supplementary Table S1 shows different MRI protocols. High resolution T2-weighted, diffusion-weighted, and T1-weighted dynamic contrast-enhanced imaging will be acquired through the MRI protocol. A radiologist (minimal 5-year experience) will describe the mpMRI findings using the PIRADS v2.1.

#### 2.4.2. TRUS biopsy

**Figure 2:**
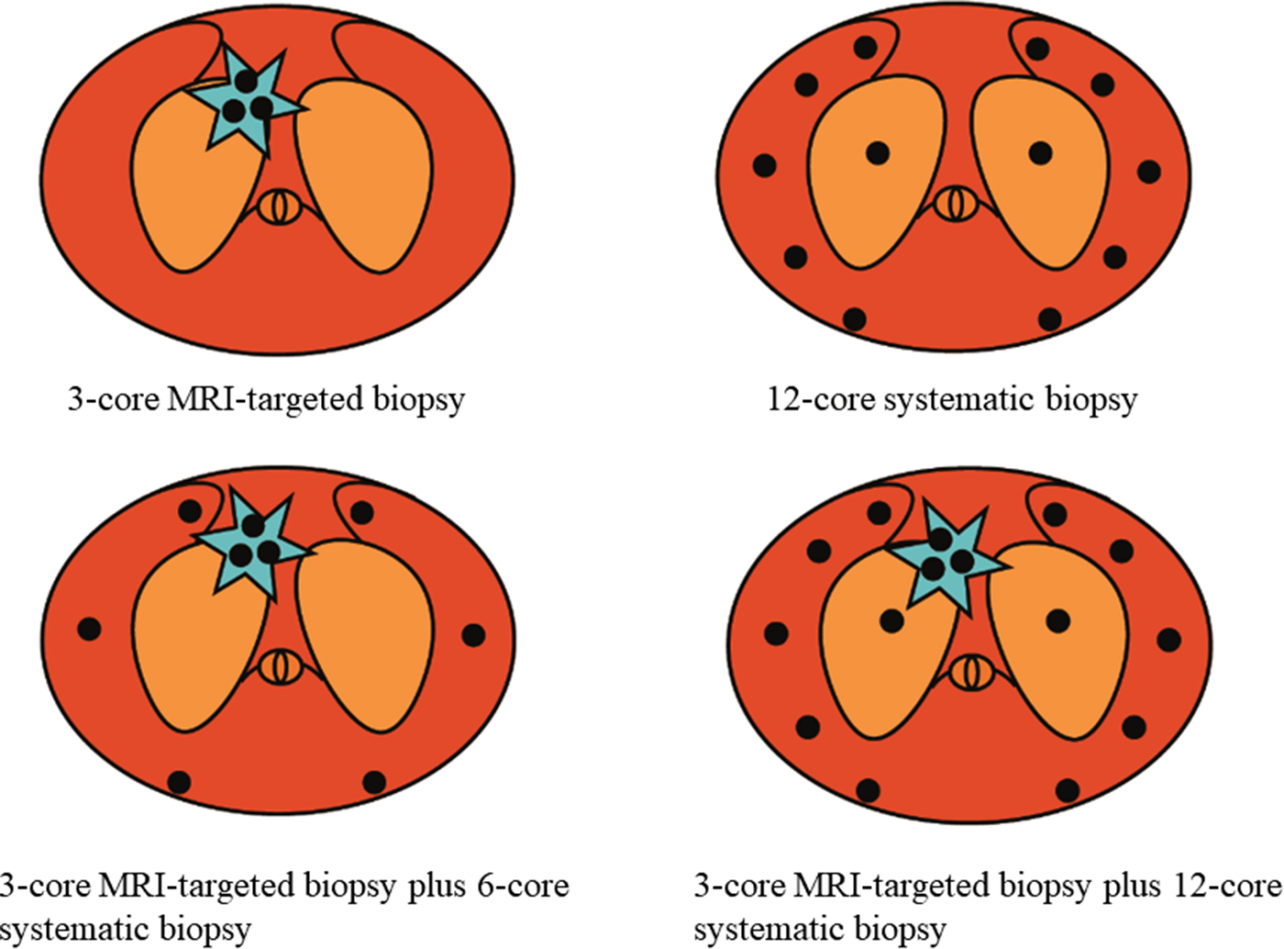
Prostate biopsy scheme.

TRUS guided transperineal prostate biopsy will be performed under local anesthesia by experienced urologists. Three cores of cognitive fusion TBx were performed within the index lesion (defined as the lesion with the highest PIRADS v2 score, or the lesion with the maximal diameter if more than two lesions with the same score). Then, 6-core or 12-core SBx will be arranged for the patients according to the enrollment (Figure 2). The standard 12- core systematic sampling protocol will be performed transperineally with five cores distributed evenly through the left and right peripheral zones, and another core will be further spread on left and right transitional zones (10). Each core will be labeled independently and sent to histology using separate containers.

#### 2.4.2. Outcome

The primary outcome compares the detection rate of PCa and csPCa between group A and group B where csPCa is defined using the Gleason scoring system (Gleason ≥3 + 4) (11). The secondary outcomes conclude as follow: (1) The yield of clinically significant prostate cancer on each side of prostate comparing it to side of lesions (contra- or ipsilateral to lesions). (2) The PCa and csPCa detection rate when adding transitional zone cores (11 and 12 cores in total). (3) The PCa and csPCa detection rate between Group A and 10-core SB plus 3-core TB in Group B. (4) The PCa and csPCa detection rate between transitional zone and peripheral zone in Group A and Group B. (5) Comparing the participant-reported pain score immediate post biopsy using pain measurement scale (Supplementary Figure S1). (6) proportion of men with post-biopsy complications and adverse events (Time frame: 7 days post biopsy, 30 days post biopsy), which include pain, fever, urinary infection/prostatitis needing antibiotics, urinary retention, erectile dysfunction, urinary incontinence, haematochezia, haematuria, and haemotospermia. (7) proportion of the men who undergo radical prostatectomy and have cancer upgraded histopathology from the biopsy to the radical prostatectomy (Table 1).

**Table 1:** Schedule of record.

	Visit 0	Visit 1	Visit 2	Visit 3
Weeks	−1	0	1	4
Teleconsult	×		×	×
Inclusion/Exclusion	×			
Family history of prostate cancer	×			
Informed consent	×			
Age	×			
t-PSA level	×			
free/total PSA ratio	×			
mpMRI result	×			
Number of lesions	×			
Size of lesions	×			
Index lesion PIRADS score	×			
Biopsy outcome			×	
Benign tissue			×	
Atypical small acinar proliferation			×	
High-grade prostatic intraepithelial neoplasia			×	
Clinically insignificant cancer			×	
Clinically significant cancer			×	
Positive biopsy cores			×	
Gleason score			×	
Men who did not undergo biopsy		×		
Undergo surgery				×
Surgery outcome				×
Pathology Gleason score				×
Immediate post biopsy questionnaire (pain)		×		
Complications		×	×	×

### 2.5. Sample size estimation

We calculated sample size according to our results of retrospective database. Based on a power of 80% and a 2.5% one-sided *α*, we used an estimate for detection rate of PCa for 6-core systematic plus 3-core MRI-targeted prostate biopsy of 61.2% and an estimate of detection rate for 12-core systematic plus 3-core MRI-targeted prostate biopsy of 64.5%. Thus, 132 men per group will be required. Accounting for a dropout rate of 10%, 280 men will need to be recruited.

### 2.6. Statistical analysis

In this study, the baseline characteristics will be summarized as the mean ± standard deviation (SD) or median (interquartile range, IQR) for continuous data and(percentages) for categorical data. We will use the univariable and multivariable logistic regression analysis to evaluate some potential predictors for csPCa (i.e., PSA density, age, prostate volume). We will use student's *t*-test for continuous variables and chi-square or Fisher exact test for categorical variables to compare the difference between group A and group B. The statistical software package R (http://www.R-project.org, The R Foundation, Boston, Massachusetts, USA) and EmpowerStats (http://www.empowerstats.com, X & Y Solutions, Inc., Boston, MA) will be used to conduct statistical analyses. All results of the analysis are considered to be statistically significant with a two-sided *P* < 0.05.

## 3. Discussion

Nowadays, it is strongly recommended that for biopsy naïve men with PIRADS score >2, combining TBx and SBx is needed. However, how to identify an ideal arrangement or permutation for systematic and TBx remains a challenge for clinicians. In other words, an ideal biopsy strategy should be developed with the goal of minimizing procedure invasion, having fewer biopsy-related complications, and maximizing the detection rate of csPCa and minimizing the detection rate of clinically insignificant cancers.

The development of systematic prostate biopsies has revolutionized over the past few decades. In 1989, Hodge et al. originally described the scheme of transrectal ultrasound guidance systematic sextant biopsies to detect prostate cancer (12); However, it could miss up to 30% of PCa (13). Then, a prospective study reported that the detection rate of PCa raised 16% when combining sextant and lateral biopsies (10 cores peripheral zone biopsies) compared to the routine sextant biopsies (194/202, 96% vs. 161/202, 80%) (14). Followed by, a 10-core biopsy scheme was developed by Gore et al. who revealed the detection rate of PCa between 10-core scheme (except for 2 mid cores from standard sextant scheme) and 12-core regional biopsy was equivalent (15). Importantly, considering the inadequacy of standard sextant biopsies, a multi-practice community based study emphasized the necessity for extended lateral side of the peripheral zone sampling (16). Therefore, in the era of SBx, the optimal sampling scheme for biopsy-naïve patients reached the consensus of a 10- to 12-cores.

With the development of mpMRI, TBx is widely accepted and related to detecting more csPCa compared to SBx. Currently, various protocols of MEI ultrasound fusion TBx were reported with 1 to 9 cores per lesion (17, 18). A consensus by the American Urological Association and Society of Abdominal Radiology guideline recommended that more than 2 cores per lesion is required for PCa detection (19). Additionally, the PRECISION trial showed that 4 cores per target to suspicious MRI lesions performed better than a standard 10- to 12-core SBx (5). More recently, Song et al. conducted a prospective trial and concluded that 3 cores per targeted lesion were suitable for transperineal MRI ultrasound fusion biopsy (20). Our previous research also proved that 3 cores per target might be the optimal biopsy scheme of MRI -targeted prostate biopsy (8).

Although targeted biopsy alone using far less biopsy cores detected no less Gleason ≥3 + 4 and fewer Gleason 6 cancers when compared with SBx, it may still inevitably underestimate a proportion of high-grade PCa in comparison with the combined approach (5, 6, 21). Previous study indicated that when using a combined SBx and targeted biopsy as a reference, directly omitting SBx resulted in detecting 13% less csPCa (7). Therefore, as strongly recommended by EAU guideline, combining targeted biopsy and SBx remains the optimal choice currently (2, 22). As previously reported, the suitable cores for the targeted and SBx have been separately investigated. Notably, how to keep the total cores (SBx plus target biopsy) lower while optimize the detection rate of csPCa is another urgent problem in the era of combined biopsy.

To optimize the sampling scheme of the combined prostate biopsy, the protocols of how to reduce biopsy cores were experimentally explored. Liu et al. divided the prostate into left, right, transitional zone, and peripheral zone, and they prospectively detected that targeted biopsy plus fewer SBx cores (nontargeted sector) had an equal PCa and csPCa detection rate as compared with the standard targeted biopsy + SBx (four cores less per patient) (23). In addition, Kachanov et al. showed that a peripheral targeted biopsy plus SBx without any transitional zone sampling detected the similar csPCa detection rate compared with the standard extended protocol (24). Furthermore, Freifeld et al. demonstrated that targeted biopsy + ipsilateral SBx may increase the detection rate of csPCa compared with only targeted biopsy, while limiting overdiagnosis of indolent PCa when comparing with targeted biopsy + contralateral SBx (25). Meanwhile, Aminsharifi et al. proposed a Reduced Core Targeted biopsy template (image—targeted + laterally directed sextant biopsy) and found that targeted biopsy + sextant biopsy could detect more csPCa patients compared to sextant biopsy; however, they did not compare the diagnostic efficiency of this sampling scheme with that of targeted biopsy + 12-core SBx (26). Shen et al. further demonstrated that targeted biopsy + lateral 6-core SBx showed the same diagnostic performance as that of targeted biopsy + 12-core SBx but with reduced cores in men with suspected PCa (27). Hence, prospective studies are warranted to provide a higher level of evidence for the optimal sampling scheme in biopsy naïve men with a PIRADS score of >2. Especially, the recent EAU guideline strongly recommended transperineal route as the preferred method considering the lower infection rates and better coverage of the anterior zone, compared to transrectal procedures (2, 22). This study is the first RCT to evaluate whether a 3-core MRI-targeted plus 6-core SBx could be considered as the optimal sampling scheme in the setting of transperineal route under local anesthesia.

There are some limitations in this study. Firstly, this trial is performed in one single high-volume center. However, we believe our preliminary findings will go a step further in providing a higher level of evidence for the optimal sampling scheme in the era of combined prostate biopsy. Secondly, the cognitive fusion mpMRI-targeted biopsies rather than ultrasound/MRI fusion software or direct in-bore guidance biopsy are performed in our institution. Nevertheless, current literatures do not report a clear superiority in detecting csPCa among these three different approaches (2), Wegelin et al. who conducted a systematic review and meta-analysis reported that no significant advantage was found between in-bore MRI target biopsy, MRI-transrectal ultrasound fusion, and cognitive registration in detecting PCa and csPCa (28). Then, Watts et al. also showed that no significant difference of PCa detection rate between MRI-ultrasound image guided fusion biopsy and cognitive biopsy (29). Thirdly, the final pathological diagnosis in both groups was based on biopsy rather than radical prostatectomy specimens which may cause potential bias. Notably, patients with negative biopsy are not likely to undergo radical therapy in clinical practice, and regular follow up after negative biopsy are also routinely scheduled in our center. Finally, we could not explore the racial disparities in our included population due to the single-center study design.

## 4. Conclusion

This protocol describes the study design and methodology of the randomized clinical trial. We aim to explore the efficacy and safety of the new biopsy strategy (6-core systematic and 3-core MRI-targeted biopsy) in biopsy naïve men with suspicious mpMRI lesion in comparison with 12-core systematic and 3-core MRI-targeted biopsy in the setting of transperineal route under local anesthesia. The expected results are meaningful, as it will provide essential data on whether decreased SBx cores could be performed in the era of combined biopsy.
