# Supplementary material for: Efficacy and safety of the new biopsy strategy combining 6-core systematic and 3-core MRI-targeted biopsy in the detection of prostate cancer: Study protocol for a randomized controlled trial
Source: Front Surg. 2023 Jan 6;9:1058288. doi: 10.3389/fsurg.2022.1058288 (PMC9852774; doi:10.3389/fsurg.2022.1058288)
Supplement: Supplementary file 1 [file Table1.doc]

**Supplementary Table 1 – Sequence parameters of multiparametric MRI protocol**

|  | Siemens | | | GE Healthcare | | |
| --- | --- | --- | --- | --- | --- | --- |
| Sequence | T2 TSE axial | EPI DWI axial | DCE axial | T2 TSE axial | EPI DWI axial | DCE axial |
| TR (ms) | 6220 | 5100 | 4.29 | 3253 | 3635 | 4.088 |
| TE (ms) | 101 | 89 | 2.14 | 129.1 | 81.5 | 1.788 |
| Flip angle (°) | 160 | 180 | 9 | 103.5 | 90 | 12 |
| Freq FOV (mm; phase FOV) | 200 | 256 | 256 | 248 | 260 | 380 |
| Slices/thickness(mm) | 3 | 3 | 2 | 4 | 4 | 2 |
| Voxel size (mm) | 0.6×0.6 | 1.6×1.6 | 0.8×0.8 | 0.6×0.6 | 1.6 × 1.6 | 0.7 × 0.7 |
| Averages/NEX | 2 | b50-2，b400-3，b800-4，b1400-6 | 1 | 2.5 | b50-2，b200-3，b1400-16 | 1 |
| b values (s/mm2; directions) |  | 50/200/800/1400 |  |  | 50/200/1400 |  |
| Time | 02:25 | 04:17 | 04:27 | 02:07 | 04:10 | 04:19 |

Abbreviations: TR = Repetition Time, TE = Echo Time, , FOV = Field of View, EPI = Echo Planar Imaging, TSE = Turbo Spin Echo, DCE = Dynamic contrast enhancement.
