# Supplementary figures and images for: Efficacy and safety of the new biopsy strategy combining 6-core systematic and 3-core MRI-targeted biopsy in the detection of prostate cancer: Study protocol for a randomized controlled trial
Source: Front Surg. 2023 Jan 6;9:1058288. doi: 10.3389/fsurg.2022.1058288 (PMC9852774; doi:10.3389/fsurg.2022.1058288)

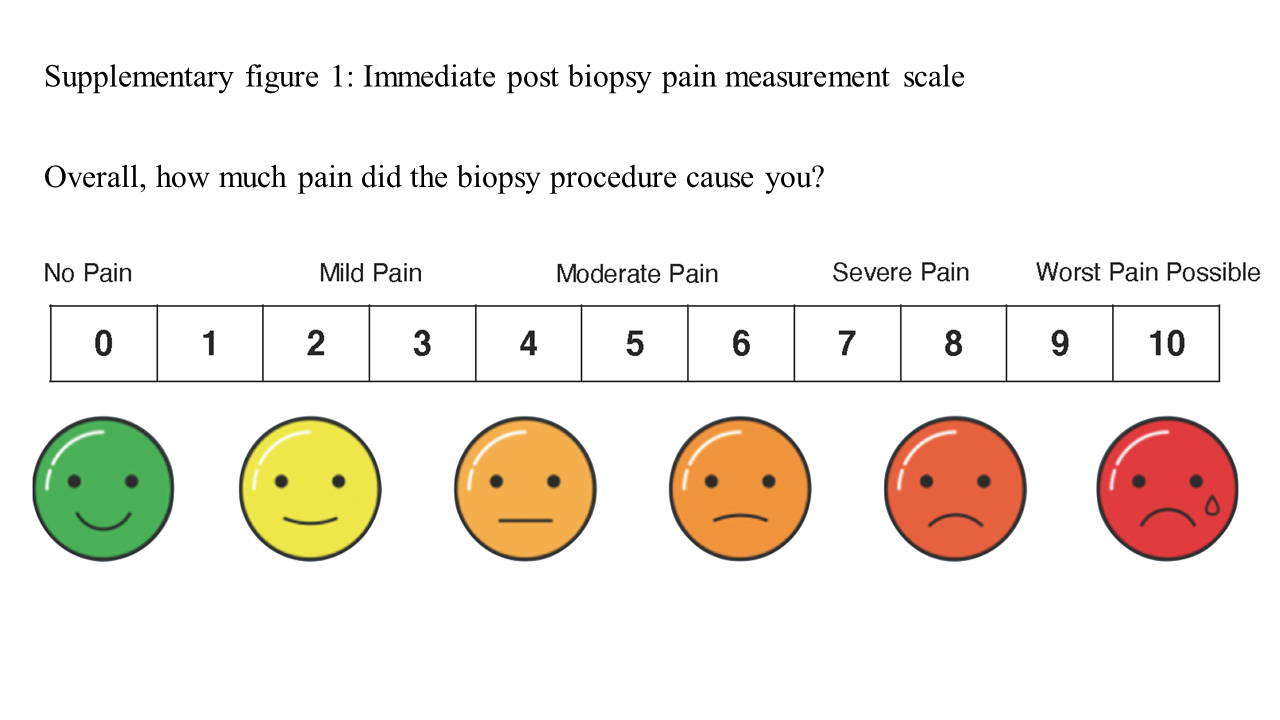

Supplement: Supplementary file 2 [file Image1.tif]
